# A New Standardized Emotional Film Database for Asian Culture

**DOI:** 10.3389/fpsyg.2017.01941

**Published:** 2017-11-03

**Authors:** Yaling Deng, Meng Yang, Renlai Zhou

**Affiliations:** ^1^Department of Psychology, School of Social and Behavior Sciences, Nanjing University, Nanjing, China; ^2^National Key Laboratory of Cognitive Neuroscience and Learning, Beijing Normal University, Beijing, China; ^3^Beijing Key Laboratory of Applied Experimental Psychology, School of Psychology, Beijing Normal University, Beijing, China

**Keywords:** emotion elicitation, emotional film database, subjective experience, physiological responses, Asian culture

## Abstract

Researchers interested in emotions have endeavored to elicit emotional responses in the laboratory and have determined that films were one of the most effective ways to elicit emotions. The present study presented the development of a new standardized emotional film database for Asian culture. There were eight kinds of emotion: fear, disgust, anger, sadness, neutrality, surprise, amusement, and pleasure. Each kind included eight film clips, and a total of 64 emotional films were viewed by 110 participants. We analyzed both the subjective experience (valence, arousal, motivation, and dominance) and physiological response (heart rate and respiration rate) to the presentation of each film. The results of the subjective ratings indicated that our set of 64 films successfully elicited the target emotions. Heart rate declined while watching high-arousal films compared to neutral ones. Films that expressed amusement elicited the lowest respiration rate, whereas fear elicited the highest. The amount and category of emotional films in this database were considerable. This database may help researchers choose applicable emotional films for study according to their own purposes and help in studies of cultural differences in emotion.

## Introduction

Emotion has always been a popular research topic, and a large range of procedures have been used to elicit emotions in the laboratory, such as pictures (e.g., Lang et al., 1999), words (e.g., Bradley and Lang, 1999), music (e.g., Sutherland et al., 1982), facial expressions (e.g., Ekman et al., 1983), imagination (e.g., Lang, 1979), films (e.g., Gross and Levenson, 1995), social interactions (e.g., Ax, 1953) and hypnosis (e.g., Bower et al., 1983). Compared to these methods the use of emotional films offers several important advantages. First, unlike slides or still photographs, films involve dynamic auditory, and visual stimuli that are complex and highly capable of capturing attention (Rottenberg et al., 2007). Second, film has a relatively high degree of ecological validity (Gross and Levenson, 1995). Third, films present more realistic emotional context whereby emotions develop over time, and allows researchers to study the time course of an emotion (Kring and Gordon, 1998). Furthermore, meta-analyses of emotion induction show that film seems to be one of the most effective ways to elicit emotions (Gerrards-Hesse et al., 1994; Westermann et al., 1996). Films can easily elicit the three main components of emotional responses: the subjective experience, behavior (including facial expression) and physiological responses (Scott, 1930; Sternbach, 1962; Averill, 1969; McHugo et al., 1982; Rottenberg et al., 2007).

Despite these advantages few film databases to elicit emotional reactions have been created. The first study using emotional films to elicit emotional reactions was done by Lazarus and his colleagues (Lazarus et al., 1962). The most commonly used standardized film set was created by Gross and Levenson (1995), which was an extension of the work of Philippot (1993) who used French dubbed film clips. Over a 5 year period they developed a set of films that reliably elicit eight different emotional states: amusement, anger, contentment, disgust, fear, neutrality, sadness, and surprise. Seventy-eight films were assessed by 494 English-speaking subjects. More recently, Schaefer et al. (2010) created another film database. They assessed 70 film clips specially developed to elicit seven types of emotion: fear, anger, sadness, disgust, amusement, tenderness, and neutrality. Development of both of these film databases relied on college students as participants, so the age range is narrow. Jenkins and Andrewes (2012) developed a new standardized film database with a wider age range of participants (18–88 years). It included 60 film clips designed to elicit six target emotions: amusement, anger, disgust, fear, happiness, and sadness, as well as a neutral state.

A more comprehensive measure of emotional response would include measures of the psychophysiological responses which are primary component of the emotion response (Scott, 1930; Sternbach, 1962; Averill, 1969; McHugo et al., 1982; Rottenberg et al., 2007). Hence, Carvalho et al. (2012) extended the previous studies by a new emotional film database that assessed both subjective experiences and physiological responses, such as skin conductance response (SCR) and heart rate (HR). They chose 52 film clips from six different categories: erotic, fear, social positive content, social negative content, scenery, and object manipulation. However, these film clips did not have sound. In a recent review Kreibig (2010) concluded many challenges remain for research on autonomic nervous system functioning using measures such as HR, respiration, and skin conductance and emotion. She noted that the heterogeneous findings could result from different paradigms to induce emotion e.g., for happiness HR is reported to increase or be unchanged with personalized recall, but also to decrease or be unchanged after viewing film clips. Furthermore, the importance of precise terminology was stressed. For example, amusement and happiness are both positive emotions but reflect different emotional responses.

One purpose of the present study is to develop a new standardized emotional movie database with audio to collect both subjective and physiological responses. It extends previous research by using Asian actors and the use of Asian participants. All the existing film databases were created by Western researchers, and have been widely used by researchers in both Western and Eastern research contexts. Some aspects of emotion are universal. Many previous studies have shown that emotional stimulus created by Western researchers can also elicit the target emotions in Asian participants (e.g., Sato et al., 2007; Zhang et al., 2013). However, the cultural differences in emotion can't be ignored. One major influence culture can have on emotion is the meaning attributed to various events. People from different cultures may appraise the same event in very different ways, depending on their own culture's system of meaning (Scherer and Brosch, 2009). Emotional responses are often influenced by culture and social environment (Kring and Gordon, 1998). For example, in the social environment where you are invited to “help yourself” at the refrigerator while being a guest in someone's home, an American might feel pleasant (an invitation to eat and you can choose whatever to eat), but a Chinese might feel unpleasant (the host will not take care of you and you can't eat anything because it's rude to take food from the refrigerator by yourself).

There is evidence from laboratory studies that shows people from different cultures have different emotional responses to the same emotional stimuli. e.g., the subjective experience for some pictures in the International Affective Pictures System (Lang et al., 1999) differs between Chinese and Western participants (Liu et al., 2009); some of the films in the database created by Gross and Levenson (1995), widely used in Western countries, do not elicit the target emotions in Asian participants (Jin, 2009). Thus, films from Western culture may elicit different types of emotions or emotional intensity in participants from Eastern cultures. The main purpose of this study was to develop an Asian emotional film database so for research on the targeted emotions in an Asian context (such as with Chinese, Japanese, Korean participants). In addition, it will allow researchers to compare emotional stimuli and emotional responses between those from Western and Eastern cultures.

## Materials and methods

### Participants and ethics

A total of 110 undergraduates and graduates (31 males, 79 females) participated in film-viewing sessions. The participants were 17–27 years old (mean age = 21.16, *SD* = 2.10), and all were Chinese. All had normal (or corrected) vision and auditory functions. The experimental procedures were approved by the Institutional Review Board of the State Key Laboratory of Cognitive Neurosciences and Learning of Beijing Normal University. All the participants gave written informed consent before participating.

### Materials

First, over a period of 6 months 8 psychology students collected samples of films with emotional content from commercial films and video clips from the Internet. Following Gross and Levenson (1995), the emotions contained in the films were sadness, anger, fear, disgust, pleasure, surprise, amusement and neutrality. From this larger pool short film clips were created by editing key sections using Format Factory software. The way we collected all the film clips were just as Gross and Levenson (1995) and Jenkins and Andrewes (2012) did. Second, 64 were selected for further evaluation based on the criteria: (1) the main actors in the films were of Asian appearance; (2) there were no visible watermarks, logos or mosaics; (3) were not cartoons (4) the thematic content was understandable without additional explanation; (5) the film elicited only one target emotion and not non-target emotions; (6) the level of arousal of emotion had to be appropriate high; and (7) the length of the film clips had to be relatively short. (The results of the target emotion and arousal rated by the psychology students in our lab were shown in the Supplementary Materials, Table S5.) There were eight films for each kind of emotions. It is worth mentioning that we distinguished amusement from pleasure strictly. The amusement films were funny to people, whereas the pleasure films showed beautiful love, best dreams, etc. We also distinguished fear from disgust strictly. There were no bloody scenes or violent fights in the disgust films. Films in this set averaged 163 s (*SD* = 80) in length (range = 58–590 s), and all had sound tracks. The details of each emotional film are shown in Table 1 (For the sources of each film please see Table S6 in Supplementary Material).

**Table 1:** Self-report ratings and psychophysiological responses for the EMDB, *mean* (*SD*).

**Emotional kind**	**No**.	**Length (s)**	**Clip description**	**Valence mean (*SD*)**	**Arousal mean (*SD*)**	**Motivation mean (*SD*)**	**Dominance mean (*SD*)**	**HR mean (*SD*)**	**RR mean (*SD*)**
Sadness	1	198	Separation of mother and son	2.519 (1.762)	5.000 (2.304)	3.778 (2.154)	4.259 (2.141)	74.333 (7.211)	14.901 (2.228)
	2	590	Brother sends younger brother and sister to others	2.833 (1.724)	5.167 (2.291)	5.367 (1.752)	4.633 (2.371)	74.793 (11.912)	
	6	154	Son knows dad die	2.500 (1.711)	5.357 (2.164)	3.786 (2.166)	4.250 (2.154)	70.082 (13.061)	15.668 (2.307)
	11	232	Aged man find himself incontinent	3.192 (1.877)	4.846 (2.292)	4.346 (2.399)	4.654 (2.038)	72.778 (10.006)	15.529 (2.970)
	12	179	Father dies in front of son	2.357 (1.545)	5.679 (2.038)	3.929 (2.523)	4.357 (2.094)	71.326 (8.988)	15.633 (2.279)
	13	179	Disabled children and unfortunate couple	3.500 (2.045)	5.333 (2.434)	5.370 (2.705)	4.296 (1.977)	72.893 (9.923)	14.967 (2.011)
	15	239	Father sends son to American	3.538 (2.044)	3.769 (2.372)	4.885 (2.215)	5.192 (2.498)	72.261 (7.965)	15.287 (1.519)
	16	234	Return objects and apologize before leaving	2.429 (1.834)	5.286 (2.665)	4.107 (2.409)	3.857 (1.938)	74.207 (11.910)	14.971 (2.167)
Anger	3	125	Don't show sympathy for disaster victims	1.393 (1.066)	5.679 (2.842)	2.143 (2.155)	5.071 (2.260)	70.803 (7.638)	15.303 (2.250)
	5	184	Hit innocent people	2.577 (1.701)	3.462 (1.985)	3.577 (1.677)	5.154 (2.327)	71.969 (8.545)	15.434 (2.308)
	6	181	Japanese bully Chinese people	2.214 (1.969)	6.071 (2.538)	4.714 (2.747)	4.714 (2.275)	74.120 (10.075)	14.918 (1.931)
	9	261	Brothers grab property after mother die	2.533 (1.613)	5.100 (2.090)	3.700 (2.575)	4.200 (2.091)	74.074 (11.188)	
	10	113	Children don't care about dead father but property	1.556 (0.974)	5.143 (2.534)	2.607 (2.283)	4.286 (2.158)	68.846 (11.782)	15.699 (2.242)
	11	146	Boss don't pay workers money	2.923 (2.058)	5.000 (2.320)	3.889 (2.190)	3.778 (2.044)	72.108 (9.406)	15.501 (2.170)
	22	180	Frame good people for money	2.346 (1.384)	4.154 (2.618)	3.538 (2.404)	5.115 (2.142)	74.014 (10.114)	16.035 (2.161)
	23	168	Passers don't save the injured girl	1.148 (0.362)	5.852 (2.741)	3.630 (2.604)	3.889 (2.391)	71.709 (8.196)	15.195 (1.987)
Amusement	5	165	Foolish robber in ancient China	6.556 (1.476)	5.963 (2.295)	5.407 (2.024)	5.519 (1.847)	71.523 (9.762)	14.564 (1.782)
	8	241	Funny competition between two teachers	5.333 (2.090)	5.600 (2.238)	4.600 (2.127)	5.033 (1.938)	72.645 (11.121)	
	9	177	Funny news	6.538 (2.353)	6.577 (1.963)	6.423 (2.403)	5.385 (2.099)	75.766 (8.441)	13.530 (2.957)
	10	196	Beautiful women seduce soldiers	5.769 (1.751)	5.615 (2.174)	5.654 (1.999)	5.731 (1.638)	72.273 (10.281)	14.889 (2.676)
	12	240	Chinese crosstalk	6.115 (2.046)	4.423 (2.101)	5.308 (2.205)	6.154 (1.515)	73.355 (8.880)	13.606 (2.077)
	13	97	Foolish robber on the train	6.964 (1.710)	6.393 (2.166)	5.926 (2.448)	5.964 (1.835)	69.618 (13.132)	14.886 (2.641)
	14	164	A rich man and a poor man sleep together	6.250 (1.956)	6.179 (2.019)	4.750 (2.784)	4.893 (2.393)	71.014 (8.177)	14.905 (1.610)
	16	225	Three officers in brothel	6.893 (1.750)	6.250 (2.102)	6.000 (2.386)	5.750 (1.936)	74.116 (13.844)	13.889 (2.406)
Surprise	1	143	An incredible fast service	5.577 (2.023)	4.231 (2.026)	5.615 (1.961)	5.385 (2.210)	73.393 (8.381)	15.219 (3.141)
	3	120	Magic	6.846 (1.759)	6.143 (2.460)	6.750 (2.319)	5.000 (2.143)	72.607 (11.373)	13.948 (2.301)
	4	99	Dominoes	7.148 (1.834)	6.556 (2.225)	7.148 (2.196)	5.741 (2.011)	72.230 (8.605)	14.597 (2.357)
	6	86	Put new steamships into ocean	5.179 (2.45)	4.25 (2.382)	4.643 (2.738)	5.321 (1.906)	73.388 (8.847)	15.476 (2.044)
	7	84	Magic in spring festival night	6.429 (1.574)	5.393 (2.25)	6.679 (1.867)	5.607 (2.347)	67.871 (11.229)	15.292 (2.758)
	12	115	Almost had a car accident but not	3.577 (2.283)	5.115 (2.613)	3.269 (2.359)	5.231 (2.338)	72.250 (10.150)	16.416 (2.434)
	13	200	Almost had a car accident but not	3.300 (2.184)	5.133 (2.300)	3.500 (2.418)	4.633 (2.526)	73.024 (11.555)	
	14	112	Peel eggs with glass cup	6.963 (1.344)	6.444 (1.625)	6.704 (1.836)	5.222 (2.136)	70.315 (7.154)	15.070 (2.139)
Fear	1	150	Ghost in the bathroom	1.889 (1.625)	6.963 (2.084)	2.556 (2.207)	3.481 (2.392)	71.861 (6.609)	15.402 (1.712)
	2	151	Meet the dead self with the ghosts	2.038 (1.685)	5.115 (2.984)	1.885 (1.532)	3.500 (2.470)	71.753 (9.903)	16.120 (2.608)
	3	83	Ghost comes out from the television	1.857 (1.433)	6.571 (2.441)	2.111 (2.359)	3.929 (2.854)	70.988 (9.224)	15.425 (2.112)
	5	156	Meet the ghost in house	2.269 (1.687)	4.462 (2.626)	2.654 (2.171)	5.038 (2.144)	71.632 (7.242)	14.357 (2.470)
	9	90	Hanged people pulled by ghost	1.370 (0.967)	5.704 (2.998)	1.556 (1.396)	3.074 (3.074)	70.907 (9.457)	15.001 (1.746)
	10	167	Ghost hides in the wig	1.800 (1.270)	6.700 (2.037)	2.690 (2.206)	4.067 (4.067)	74.797 (12.775)	
	11	80	A girl in a dark house	1.778 (1.281)	6.214 (2.470)	2.321 (2.229)	3.464 (3.464)	69.845 (13.871)	15.972 (1.763)
	12	153	Ghost under the bed	2.143 (2.121)	6.857 (2.337)	2.786 (2.558)	3.536 (3.536)	74.294 (13.841)	14.786 (2.172)
Disgust	3	68	Rack acne	2.074 (1.730)	4.704 (2.998)	2.037 (1.911)	3.000 (1.754)	74.686 (11.272)	14.404 (2.031)
	4	165	Food vomited from the stomach	1.192 (0.801)	4.500 (3.444)	1.346 (1.346)	4.231 (2.643)	74.358 (8.523)	14.873 (2.211)
	6	123	Put crawling maggots on face	1.769 (1.531)	5.308 (2.881)	1.846 (1.690)	3.846 (2.378)	72.403 (9.087)	15.822 (2.597)
	7	60	Dirty food material	1.536 (1.688)	5.643 (2.297)	2.536 (2.603)	4.750 (2.319)	72.703 (9.155)	14.609 (2.742)
	9	87	A man vomits	2.923 (2.331)	5.615 (2.368)	1.500 (1.105)	4.346 (2.513)	72.872 (5.628)	14.963 (1.927)
	11	169	Dirty and smelly foot	3.267 (3.267)	5.600 (1.886)	3.800 (2.747)	5.000 (2.101)	72.094 (9.410)	
	17	58	Cockroach is crushed	1.964 (1.575)	5.750 (2.413)	2.593 (2.422)	4.036 (2.269)	69.322 (13.360)	15.028 (2.277)
	18	71	Pustule on body	1.143 (0.448)	6.000 (3.151)	1.500 (1.753)	2.929 (2.308)	75.948 (12.723)	13.691 (2.410)
Pleasure	1	219	Children play on grass	7.615 (1.169)	5.731 (2.219)	7.192 (1.789)	5.385 (2.137)	72.837 (10.191)	13.896 (1.684)
	2	259	Lovers chat happily	7.000 (1.912)	5.200 (2.041)	6.333 (2.279)	5.333 (2.023)	73.926 (10.982)	
	5	100	Lovers chat about sex	7.444 (1.476)	5.444 (2.118)	5.889 (2.577)	5.148 (1.586)	73.124 (9.959)	14.802 (1.583)
	11	129	A man gives gifts to a woman and says love to her	7.769 (1.107)	5.462 (2.319)	6.846 (1.515)	6.346 (1.623)	72.100 (9.280)	15.571 (2.756)
	13	125	Lovers talk about the rainbow	6.704 (2.035)	4.889 (2.293)	6.222 (1.928)	5.556 (5.556)	73.047 (6.587)	15.172 (1.860)
	14	209	Happy time of father and son	8.607 (0.497)	6.929 (1.631)	8.357 (0.678)	6.321 (1.867)	74.324 (10.101)	14.266 (2.893)
	15	79	Happy time in high school	8.000 (1.333)	6.929 (1.654)	7.643 (1.420)	5.964 (2.027)	70.325 (11.392)	15.004 (2.604)
	16	97	The aged people go hiking	7.429 (1.773)	6.179 (2.262)	7.321 (2.278)	5.000 (1.981)	72.149 (8.337)	15.320 (2.804)
Neutrality	1	181	City in heavy snow	4.074 (1.615)	2.444 (2.444)	4.148 (1.975)	5.185 (1.570)	75.042 (8.028)	15.326 (2.020)
	2	244	City in snow	4.120 (1.666)	2.385 (1.602)	3.885 (1.862)	5.577 (2.248)	74.241 (10.958)	14.565 (2.008)
	3	347	Talk about the relationship between weather and red leaves	4.250 (1.818)	1.929 (1.274)	3.821 (2.144)	4.679 (2.480)	74.949 (12.05)	14.535 (1.864)
	4	110	Talk about the recovery from drought	3.714 (1.740)	2.321 (1.657)	3.714 (1.652)	5.536 (1.895)	75.901 (9.548)	15.010 (2.592)
	6	153	A man teaches how to drive	3.867 (1.995)	3.200 (2.265)	4.733 (2.318)	5.067 (2.504)	73.819 (8.216)	14.754 (1.192)
	14	150	Drive on the mountain road	4.037 (1/786)	2.963 (1.652)	3.741 (1.655)	4.444 (2.242)	74.594 (7.715)	15.493 (1.830)
	17	180	A man teaches calligraphy	5.929 (1.884)	4.214 (4.214)	5.821 (2.109)	5.357 (1.948)	71.100 (10.992)	15.493 (2.274)
	18	180	Weather forecast	4.692 (2.187)	2.538 (1.655)	4.654 (1.938)	5.923 (2.038)	75.913 (11.148)	

### Procedure

The participants performed the task in a room under similar lighting conditions one by one and were seated at a 90°-angle arc facing the screen. The films were displayed using a 14-inch computer screen. Prior to viewing the films, the participants signed a consent form and answered several demographic questions. The experimenter stated that the purpose of the study was to learn more about emotions. Participants were told that the films would be shown on a computer screen and they should watch the film carefully but could look away or shut their eyes if they found the films too distressing or could stop the experiment if they felt uncomfortable at any time.

We collected data on both the subjective experience (described below) and physiological responses. Heart Rate (HR) and Respiration Rate (RR) are currently two of the most common autonomic nervous system markers of emotional processing (see Kreibig, 2010, for a review; Mauss and Robinson, 2009 for a review). The HR and RR data were collected on a BIOPAC MP150 system with AcqKnowledge 4.0 (BIOPAC Systems Inc.). HR was assessed using a 3 lead ECG, with a lead II configuration. RR was measured by applying a rubber band around the chest. Data were analyzed offline using AcqKnowledge 4.0 software (BIOPAC Systems Inc.).

Before watching the films, participants were asked to rest for 2 min as a baseline. Prior to each film, participants were shown a blank screen for 30 s and instructed to “clear your mind of all thoughts, feelings, and memories”. While watching, participants were asked try to move as little as possible to ensure the quality of the HR and RR data. After each film, participants were asked to complete a 28-item self-report inventory which participants rated Valence, Arousal, Dominance, Motivation, Familiarity, Likability, Amusement, Anger, Confusion, Disparagement, Pleasure, Disgust, Embarrassment, Fear, Happiness, Interest, Pain, Extrication, Sadness, Surprise, Tenseness, Shame, Guilt, Repentance, Compunctious, Alertness, and Concentration. The last item was to ask the participants choose one word from the 21 items (except the valence, arousal, motivation, dominance, familiarity and likability) which best described the type of emotion of this film. This rating procedure, adapted from Ekman et al. (1980), was similar to that used by Gross and Levenson (1995). It was a 9-point Likert scale. For the familiarity and likability items the anchor points of 1 and 9 correspond with “not at all” and “very much,” respectively.

Participants rated valence, arousal, motivation and dominance using the paper version of the Self-Assessment Manikin (Hodes et al., 1985). For Valence the anchor points were 1 for “very unhappy” and 9 for “very happy;” for Arousal 1 for “very calm” and 9 for “very aroused;” For Motivation 1 for “high desire to withdrawal” and 9 for “high desire to approach;” and for Dominance the rating was 1 for “feeling totally dominated” and 9 for “feeling strong control power.” For the other items, 1 indicated “did not feel even the slightest bit of the emotion” and 9 indicated “feel a lot of the emotion” (see (Lang et al., 1999) for a detailed explanation). There was a 30 s delay to clear the mind before the next film was presented.

Each participant viewed 16 films (2 films per kind) and each film was viewed by 28 participants. The trial order was designed so that (1) no two films targeting the same emotion were shown in a row, (2) no more than three films of a particular valence (negative or positive) were shown consecutively and (3) each film had the same chance to be shown in every order for different participants.

### Data analysis

The mean score and SD for each kind of emotion were calculated. Six separate within-subject repeated measures of ANOVA with eight levels (sadness, anger, amusement, surprise, fear, disgust, pleasure, neutrality) were performed for the valence, arousal, motivation, dominance, heart rate and respiration rate data. All the *post-hoc* multiple pairwise comparisons were performed using Bonferroni's correction. The level of statistical significance was set at *p* < 0.05. Data analyses were performed using IBM PASW Statistics 18.0 (IBM).

## Results

### Self-report ratings

**Figure 1:**
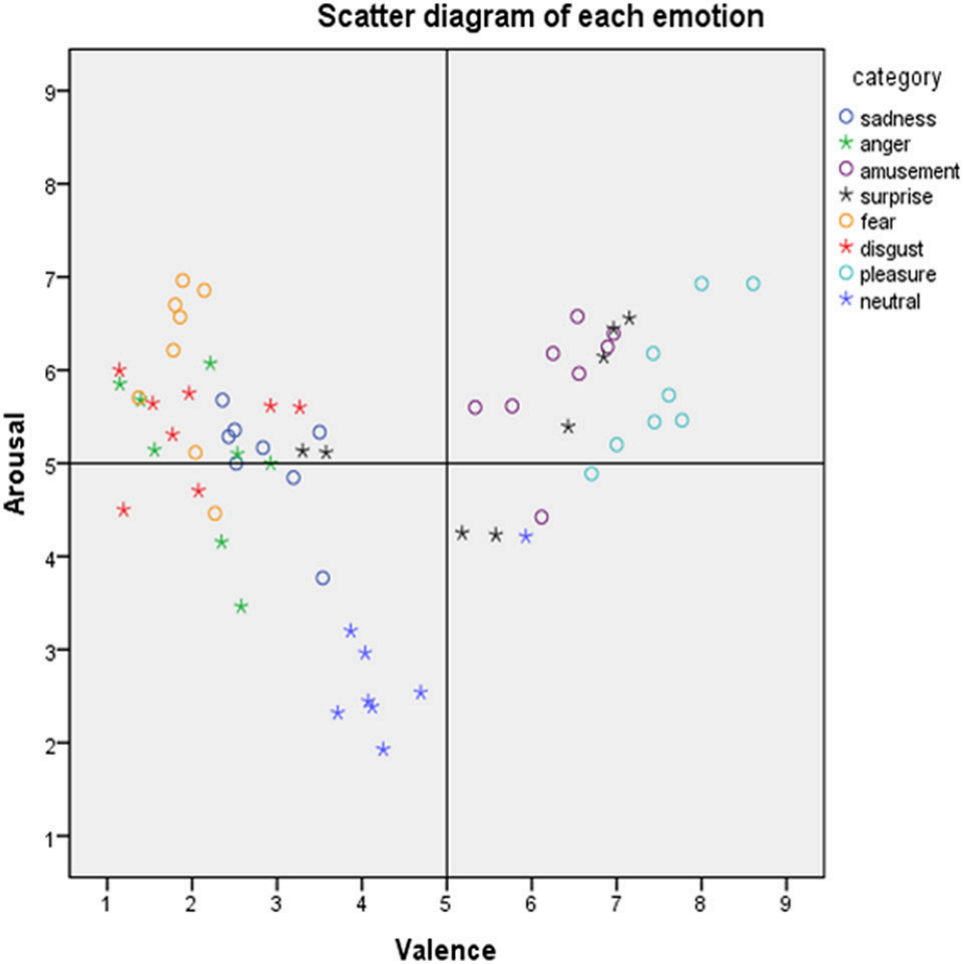
The distribution of the film clips in the affective space (mean values for valence on the horizontal axis and arousal on the vertical axis).

**Figure 2:**
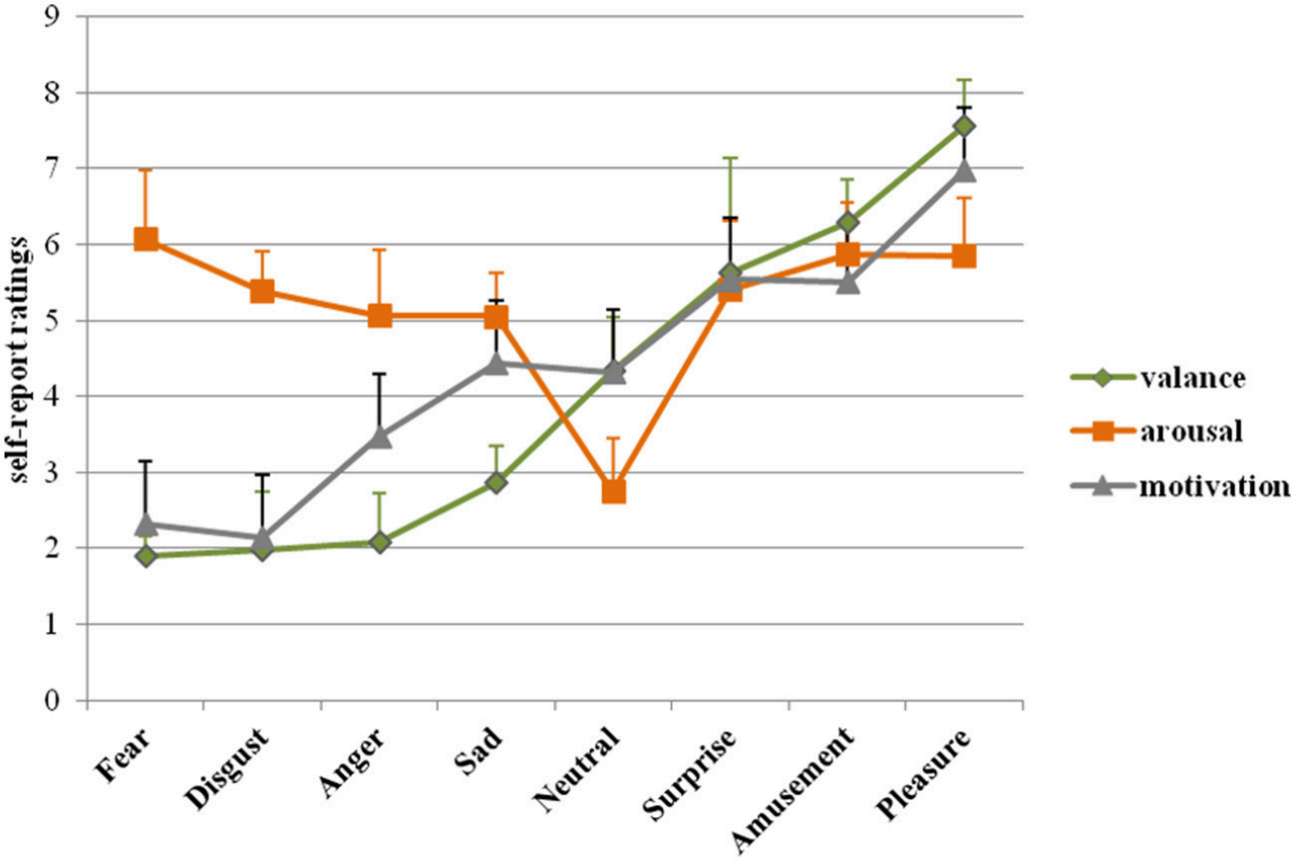
The rank of each category on valence, arousal and motivation. (The rank is based on the results of valence.)

Table 1 presents a general description of the film clips, with the associated self-report ratings of valence, arousal, motivation, and dominance. The results of other 24 items are shown in the Supplementary Materials, Table S6. All 64 films are included. We asked the participants use one word to describe the type of emotion of each film. We counted the number of times each word was used as a descriptor as the hit rate. The total hit rate was 99%, which showed that almost all the participants gave the target word. This result demonstrated the effectiveness of the classification and that the films elicited the target emotions. Table 2 presents a general description of per category. Figure 1 shows the distribution of the ratings of valence and arousal. Figure 2 shows the rank of each category on valance, arousal and motivation.

**Table 2:** Self-report ratings and psychophysiological responses per category, *mean* (*SD*).

**Film category**	**Valence mean (*SD*)**	**Arousal mean (*SD*)**	**Motivation mean (*SD*)**	**Dominance mean (*SD*)**	**HR mean (*SD*)**	**RR mean (*SD*)**
Sadness	2.859 (0.488)	5.055 (0.576)	4.446 (0.673)	4.437 (0.394)	74.100 (11.452)	15.202 (2.064)
Anger	2.086 (0.640)	5.058 (0.880)	3.475 (0.786)	4.526 (0.561)	73.580 (10.683)	15.377 (1.830)
Amusement	6.302 (0.556)	5.875 (0.682)	5.508 (0.624)	5.554 (0.437)	72.954 (10.413)	14.388 (2.041)
Surprise	5.627 (1.514)	5.408 (0.912)	5.538 (1.551)	5.268 (0.345)	73.221 (12.763)	15.041 (2.163)
Fear	1.893 (0.273)	6.073 (0.904)	2.320 (0.437)	3.761 (0.599)	73.067 (11.889)	15.378 (1.839)
Disgust	1.984 (0.767)	5.390 (0.525)	2.145 (0.816)	4.017 (0.747)	74.588 (13.613)	14.717 (1.948)
Pleasure	7.571 (0.587)	5.845 (0.766)	6.976 (0.819)	5.632 (0.519)	74.216 (11.677)	14.921 (2.116)
Neutrality	4.335 (0.705)	2.749 (0.709)	4.315 (0.728)	5.221 (0.487)	75.538 (11.914)	14.931 (1.772)

#### Valence effects

A one-way repeated measure ANOVA revealed significant differences between the valence ratings [*F*_(7, 756)_ = 296.648, *p* < 0.001, η^2^ = 0.733] of the films clips. *Post-hoc* Bonferroni pairwise comparisons indicated that there were no significant differences between the film clips with negative emotions (anger, fear, and disgust) but they were significantly different from the other kinds. All the valence ratings of the positive films were significantly higher than those of neutral films, and those of neutral films were significantly higher than those of the negative films. Further, the other kinds differed significantly from each other. The valence ratings were as follows: anger, fear, disgust < sadness (*p* < 0.001) < neutrality (*p* < 0.001) < surprise (*p* < 0.001) < amusement (*p* < 0.001) < pleasure (*p* < 0.001). In addition, pleasure was rated more positively than amusement.

#### Arousal effects

A one-way repeated measure ANOVA revealed significant differences among the arousal ratings [*F*_(7, 756)_ = 50.482, *p* < 0.001, η^2^ = 0.319] for the different film clips. *Post-hoc* Bonferroni pairwise comparisons indicated that arousal for neutral film clips was significantly lower than that of any other categories (all *p* < 0.05). Films depicting sadness and anger were rated as less arousing than pleasure, amusement and fear (all *p* < 0.05). Furthermore, disgust was rated as less arousing than fear (*p* < 0.01). There were no significant differences between the other comparisons.

#### Motivation effects

A one-way repeated measure ANOVA revealed significant differences among motivation ratings [*F*_(7, 756)_ = 100.050, *p* < 0.001, η^2^ = 0.481]. *Post-hoc* Bonferroni pairwise comparisons indicated that the motivation of these eight kinds of films were as follows: disgust, fear < anger (*p* < 0.001) < neutrality, sadness (*p* < 0.05) < amusement, surprise (*p* < 0.001) < pleasure (*p* < 0.001). Positive films (such as pleasure, amusement, and surprise) elicited approach motivation, and negative films (such as sadness, anger, fear, and disgust) elicited avoidant motivation. It is worth mentioning that neutral films elicited avoidant motivation as sad.

#### Dominance effects

A one-way repeated measure ANOVA revealed significant differences among dominance ratings [*F*_(7, 756)_ = 24.494, *p* < 0.001, η^2^ = 0.185]. *Post-hoc* Bonferroni pairwise comparisons indicated that there were no significant differences between three negative emotions (fear, disgust and sadness) and no differences between three other negative emotions (disgust, sadness and anger); however, the dominance of fear was significantly lower than that of anger (*p* < 0.05). The dominance of positive (amusement, surprise and pleasure) and neutral emotions were significantly higher than that of negative emotions (*p* < 0.05). There were no significant differences between neutrality, surprise, amusement, and pleasure.

#### Target emotions

The results of mean ratings for target emotion were showed in Table 3. Target emotion means the emotion which was elicited most strongly. For example, for sadness films, the rating of sadness was the rating of target emotion. One-way repeated measure ANOVA revealed significant differences between the ratings of target emotion and non-target emotion, all *p* < 0.05.

**Table 3:** Ratings of target emotion per category, *mean* (*SD*).

**Film category**	**Sad**	**Angry**	**Amuse**	**Surprise**	**Fear**	**Disgusting**	**Pleased**
Sadness	6.291 (0.750)	1.757 (0.536)	1.233 (0.181)	1.661 (0.276)	1.584 (0.270)	1.592 (0.284)	1.532 (0.376)
Anger	3.978 (1.469)	6.626 (0.633)	1.309 (0.258)	2.165 (0.733)	1.982 (0.733)	5.323 (0.876)	1.287 (0.210)
Amusement	1.130 (0.109)	1.394 (0.186)	6.955 (0.642)	1.860 (0.471)	1.272 (0.166)	1.664 (0.461)	4.163 (0.471)
Surprise	1.192 (0.211)	1.318 (0.301)	2.590 (0.466)	5.092 (0.935)	1.781 (0.859)	1.759 (0.632)	3.798 (1.193)
Fear	1.721 (0.265)	1.783 (0.281)	1.253 (0.176)	2.584 (0.316)	6.745 (0.468)	4.612 (0.542)	1.277 (0.211)
Disgust	1.937 (0.430)	2.961 (1.729)	1.845 (0.856)	2.941 (0.664)	3.833 (1.502)	6.315 (1.219)	1.479 (0.364)
Pleasure	1.255 (0.308)	1.147 (0.147)	2.968 (1.416)	1.795 (0.526)	1.134 (0.169)	1.223 (0.254)	6.597 (0.871)
Neutrality	1.161 (0.189)	1.191 (0.242)	1.230 (0.149)	1.701 (0.570)	1.196 (0.263)	1.692 (0.339)	2.176 (0.669)

Table 4 shows a comparison of the results from the present study with that of Gross and Levenson (1995). Our data are the mean ratings for target emotion of the eight kinds of emotional film clips (see Table 3). For Gross and Levenson (1995) the mean ratings was of their best two films for each category (see their Table 1). Because we used a 1–9 point scale and Gross and Levenson used a 0–8 point scale we subtracted 1 point of all the results before the comparison. The results in Table 4 showed the results after subtraction of our results.

**Table 4:** Ratings of target emotion compared this database with Gross and Levenson (1995).

	**Sadness**	**Anger**	**Amusement**	**Surprise**	**Fear**	**Disgust**	**Pleasure**
This database	5.291	5.626	5.955	4.092	5.745	5.315	5.597
Gross's	5.530	5.635	5.700	4.635	4.160	6.025	3.520

### The psychophysiological responses

Table 1 presents the psychophysiological responses of heart rate and respiration rate of each film clip. Table 2 presents a general description of per category.

#### Heart rate (HR)

A one-way repeated measure ANOVA revealed significant differences in the heart rate data [*F*_(7, 756)_ = 4.072, *p* < 0.001, η^2^ = 0.036]. *Post-hoc* Bonferroni pairwise comparisons indicated that the HR for neutrality film clips was significantly higher than those of sadness, anger, amusement, surprise and fear (all *p* < 0.05). There were no other differences.

#### Respiration rate (RR)

For one group the RR data was lost due to equipment failure. Thus, there were missing data in each category of film clip (see Table 1).

A one-way repeated measure ANOVA revealed significant differences in the respiration rate data [*F*_(7, 455)_ = 5.204, *p* < 0.001, η^2^ = 0.074]. *Post-hoc* Bonferroni pairwise comparisons indicated that the RR for amusement was significantly lower than those of sadness, anger and fear (all *p* < 0.05), and disgust was lower than fear (*p* < 0.05). There were no other significant differences.

### Length effect

Because the length of each film clip was different (from 58 to 590 s), we further analyzed whether the length factor could induce different effects in inducing valence, arousal, motivation, dominance, HR and RR. We used the median (155 s) as the boundary. The length shorter than 155 s were sorted as short length, the length longer than 155 s were sorted as long length. Independent sample *T*-test was used to test the length effect within each kind of emotion. The significance level was *p* < 0.001 after the Bonferroni's correction for multiple pairwise comparisons. The results only showed the signicant differences in surprise emotion. Short length of surprise emotional films elevated both higher valence (short: 5.968 ± 2.235, long: 3.300 ± 2.184) and motivation (short: 5.847 ± 2.533, long: 3.500 ± 2.418) than long length. There were no significant differences between short and long length in other kinds of emotional films in inducing valence, arousal, motivation, dominance, HR, and RR.

## Discussion

The present study showed the development of a new emotional film database with Asian actors where both the subjective (valence, arousal, motivation, and dominance) and physiological responses (HR and RR) of Asian participants were measured. It consisted of film clips with audio which depicted eight kinds of emotion (fear, disgust, anger, sadness, neutrality, surprise, amusement, and pleasure) similar to previous database studies (Gross and Levenson, 1995; Schaefer et al., 2010; Carvalho et al., 2012; Jenkins and Andrewes, 2012). The data (Table 4) showed the ratings of the target emotion in this film database were similar to Gross and Levenson (1995). Thus, it comprised a relatively well-developed standardized emotional film database. In addition, each category consisted of eight films, and each film in the same emotion had different scores on valence and arousal (see Figure 1), so researchers can select varying degrees of emotional films according to their own needs.

Self-reports from the participants indicated that each film clip elicited the target emotion (see Tables 1, 3). As expected fear, disgust, anger, and sadness elicited negative emotions. Surprise, amusement and pleasure elicited positive emotions. The valence of neutral film clips was rated in the middle of the scale (mean score close to 5) and the arousal of neutrality was the lowest (almost < 3), indicating some emotional neutrality. These data suggest the neutral films may be particularly appropriate for use as a control category.

For the valence rating, participants rated film clips of anger, fear, and disgust significantly lower than those depicting neutrality and positive emotions. However, within the negative emotions there were no differences between each other, suggesting the three kinds of emotional film clips in this database made participants experience the same feeling of unpleasantness. In contrast, the ratings of valence between film clips depicting positive emotions did differ: pleasure had the highest valence, pleasure films clips indeed made people feel happier than the amusement films clips, and surprise was rated less positive than that of amusement. Though the pleasure and amusement emotions were both positive, the content of the emotional stimulus may play more important role (Carvalho et al., 2012). As for the surprising emotion, they were rated as “Pleased.” However, surprising emotion is well-known for its ambiguous property (Kim et al., 2004). It has to be clarify that the surprising emotional film clips in the present study showed the unusual things, for instance, magic, and uncredible skill. This kind of emotion was adapted from Gross and Levenson (1995). People may feel ambiguous at the beginning, but after they knew the principle they may suddenly realized and feel amazing and pleased. So the surprising emotion in this study was a little different from the studies about surprising expressions. Thus, the research on emotion needs to consider more than valence; rather, the content of the emotional stimulus should be taken into account.

The arousal dimension has been conceptualized as an index of the intensity of an emotional reaction (e.g., Schupp et al., 2004). In the present study neutral films had the lowest arousal compared to any other categories. Fear, amusement, and pleasure had higher levels of arousal than the sadness and anger films, but unlike Carvalho et al. (2012) and Lang et al. (1999), pleasure, amusement and fear did not differ from each other. This finding that film clips depicting sadness and anger film clips did not elicit higher levels of arousal than positive ones may be accounted for cultural differences. According to many cultural psychologists, people in Western cultures (especially Americans) tend to be high on individualism and in contrast, most Asian cultures emphasize collectivism, or prioritizing the group over the individual (Markus and Kitayama, 1991). For example, one study about Chinese Americans reported that less acculturated Chinese Americans speak significantly more than the others did about friends, family, and other social activities (Tsai et al., 2004). Positive film clips in this study all included happy interactions between lovers, family members, or friends. These stimuli may make people in collectivism cultures feel the greater happiness, resulting in higher ratings for arousal of positive emotions. This explanation would account for the difference in findings with previous research and the lack of difference in measures of arousal between negative and positive emotions in this study. Importantly the data suggest it is possible to produce film clips which elicit same levels of arousal for positive and negative films, removing a possible confound from research on emotions.

The third dimension measured was dominance, somewhat neglected, due to its variance being the least unique (e.g., Bradley and Lang, 1994) within the affective space, but it is worth measuring for further investigation (Carvalho et al., 2012). Bradley and Lang (1994) stated that the dominance dimension sometimes may be confusable. The main problem is what is really being rated: the stimulus itself or the subjective feeling it elicits. To avoid this confusion, in the present study the instructions to participants emphasized the elicited feeling. A lower degree of dominance represented that participants felt more dominated by the content of the emotional films. The positive and neutral movies had larger dominance scores than the negative ones. For the negative films, the fear clips had lower scores compared to anger. In short, participants reported higher levels of being dominated appear after negative films, especially fear ones, consistent with what was found in previous studies of emotional film databases (Carvalho et al., 2012). However, this finding is inconsistent with that from a study of affective pictures (Bradley and Lang, 2007), further emphasizing that variations in paradigms need to be accounted for in interpreting the results of studies of emotion.

This study also measured motivation, another under-studied variable in previous studies of emotional film databases. Links between emotion and the motivation system have been studied (Elliot et al., 2013). Emotions can activate the approach or avoidance motivation systems (e.g., Lang et al., 1990, 1999). The data in the present study speak to this issue. Consistent with previous research (e.g., Frijda, 1988; Rolls, 2013; Panksepp, 2015) in this study, the negative emotion film clips (except sadness, which showed the same motivation as the neutral film clips) elicited the avoidance motivation (with motivation scores lower than 5) and the positive ones elicited the approach motivation (with motivation scores higher than 5). Like in the valence data, the film clips for pleasure and amusement were rated differently such that pleasure film clips elicited more approach motivation than amusement films. In contrast to the finding for the valence data, for the motivation scores there were reliable differences between film clips portraying negative emotions: sad films had higher motivation scores than anger films, and anger films had higher scores than disgust and fear films. One interpretation of this complex data set is that sadness may make people show more sympathy, so sad movies had low valence but high motivation scores. Anger is a negative emotion, but it often elicited the approach motivation (Harmon-Jones, 2003, 2004a,b); consistent with data from this study which showed that anger film clips also had low valence but high motivation scores.

One strength of this study was concurrent measurement of subjective and psycho-physiological responses. HR was significantly higher when participants viewed neutral emotional film clips than when viewing those depicting sadness, anger, amusement, surprise, and fear. That is to say, compared to neutral films, HR declined while watching emotional films. This finding is consistent with some previous results (Bradley et al., 2001; Sánchez-Navarro et al., 2006; Codispoti et al., 2008; Carvalho et al., 2012) but not with others (e.g., Kunzmann and Grühn, 2005 found the increased HR for sad movies in aged people). Kreibig (2010) has reviewed many studies about autonomic nervous system activity in emotion and found the great diverge of autonomic response patterns for certain emotions. She discussed each kind of emotions detailed in the review and found “HR was increased for negative (anger, anxiety, contamination related disgust, embarrassment, fear, crying sadness) and positive emotions (imagined anticipatory pleasure, happiness, joy) as well as for surprise. HR decreased in mutilation-related disgust, imminent-threat fear, non-crying sadness, acute sadness, affection, contentment, visual anticipatory pleasure, and suspense emotions that all involve an element of passivity, and may be taken to suggest vagal mediation.” In consideration of these inconsistent findings, we thought the content of the emotional stimulus but not just the valence or arousal may play an important role in the physiological responses. Besides, different operations of the experiment (age and gender/sex of participants, length of stimulus, time of day/year of the test, etc.) may also be responsible for the diverge. Numerous conclusions remain tentative and more studies are still needed. We hope this database could offer more stimulus for future researchers to further explore the autonomic nervous system activity in emotion. To explain the results in this study, we would like to refer to Codispoti et al. (2008). They proposed that a decline in heart rate reflected orienting, sustained attention and action preparation, consistent with the finding that in the present study neutral film clips were less arousing than each of the emotion inducing film clips.

Few studies of emotional stimulus database have measured respiration rate. The respiration rate means how deeply and rapidly a person is breathing. In this study, amusement films elicited the lowest respiration rate, whereas fear elicited the highest. The fear, anger, and sadness films elicited higher RRs than the amusement films. Disgust films elicited lower RRs than fear films. The fear films made people breath quickly and deeply, indicating tense anticipation. These results were consistent with previous studies (Haag et al., 2004). Rapid and deep breathing can indicate excitement, such as anger or fear, but sometimes also joy. Rapid shallow breathing can indicate tense anticipation, including panic, fear or concentration. Slow and deep breathing indicates a relaxed resting state, whereas slow and shallow breathing can indicate states of withdrawal, passive states, such as depression or calm happiness (Haag et al., 2004).

The films in this database were all from Asian culture, purposely constructed to be a valid measure of emotional constructs in that cultural context. No erotic stimuli were included in any of the clips designed to elicit positive emotions. While in Western culture, erotic pictures or films have been used to elicit positive and high-arousal emotions (e.g., Carvalho et al., 2012), participants from Eastern culture have been reported to rate erotic stimulus as less arousing and less pleasant (Liu et al., 2009). In fact, Chinese participants rated the valence of naked people and sex lower than neutral stimulus (Liu et al., 2009). However, Eastern people rated warm relationships with others (e.g., clips No. 14–16 of pleasure stimuli) as the most positive and arousing. This category is often rated as a positive but low-arousal emotion in Western culture. One possible explanation for this difference is the collectivism in Eastern culture and individualism in Western culture (Earley, 1993). As for the fear emotion, the things that made people feel fear were totally different between the cultures. The fear film clips in this study excluded bloodiness, murder, and violence which sometimes make Eastern people feel disgusted rather than fearful. Rather we included clips from classical Asian ghost films, such as “A Wicked Ghost,” as fear films. Although the content of fear films was different between that used in Western data bases, fear films were still rated the most negative and highly arousing emotion compared to other emotions in both cultures. In short, some types of emotional films created by Western researchers were not suitable for eliciting the same emotion or emotional intensity in Eastern participants. As a result, this database was developed.

Finally, there are some limitations that should be considered with caution. First, the HR and RR may not be enough to represent complex physiological patterns of nervous systems. Second, the length and the exciting parts of the films were different. This may bring about the potential problem that the time course of the emotion could not be easily determined. For example, the most exciting part of one film clip was at the end, and the physiological responses changed only at the end. However, the average physiological responses may be affected by the first half of a film clip. Third, the sample of participants in this study was small, with a restricted age range (17–27 years old), they may not representative of the overall population, with a preponderance of females. Given known sex differences in emotion (Fischer et al., 2004), this may affect the results, though several previous studies also had this gender distribution (Schaefer et al., 2010; Carvalho et al., 2012). We have to say the current study can't be considered a normative study due to sample limitations. The present data requires replication, with larger samples and including, for instance, different range of ages, different nationalities as mentioned below, socioeconomic status and so on. Fourth, though the films were chosen from several Asian countries, the participants in this study were Chinese. Though studies found few or no cultural differences in emotional perception between Japanese and Chinese participants (Kawamura and Ohno, 2011), other researchers found that Japanese today are about as individually competitive as Americans (Bond, 2002; Oyserman et al., 2002). We still need more studies to test the cultural differences among Asian countries. The limitation of using films to elicit emotions has been discuss extensively (e.g., Fernández et al., 2012). Future studies could include (1) considering brain activity while watching these films, (2) enlarging the sample of participants in the nationalities, for example, age range, since huge number of researches have found that older people have a different approach to emotional stimuli compare to young people (e.g., Mammarella et al., 2012, 2016; Fairfield et al., 2013; Di Domenico et al., 2015, 2016), (3) using other methods to measure the autonomic nervous system, and (4) Comparing the differences in emotional responses between Western and Eastern culture. The current results were soley based on Asian population and therefore additional data based on Westerners should be collected to valid the usefulness of this dataset compared to the previous Western based stimulus sets. We just provide the stimulus in this study and we will collect more data using these stimulus. We also hope more researchers can use these stimulus to do more comparative studies, just like IAPS (Bradley and Lang, 2007), and this dataset can be used to compare research across laboratories or simply as a source of experimental materials.

## Conclusion

The amount and category of emotional films in this database were considerable. The positive ones and negative ones had the same level of arousal, which can activate appetitive and defensive systems equally. This database may help Eastern researchers choose applicable emotional films for research. There were both subjective experience and physiological response in this database. Each category contained eight film clips and the eight films had different parameters from which researchers can choose, such as high and low arousal of pleasure. In addition, this database may be helpful for studies of cultural differences in emotions.

## Author contributions

RZ and YD designed the study, MY and YD collected and analyzed the data, YD and RZ wrote the manuscript.

### Conflict of interest statement

The authors declare that the research was conducted in the absence of any commercial or financial relationships that could be construed as a potential conflict of interest.

## Supplementary material

The authors of the current database wish to state that they do not make any claim over the copyright of the film clips. That copyright is exclusively of the films rightful owners, which can be found in the Table S6 of supplementary material. The database film clips described in this article can be only used for scientific research and may not be used for any commercial purposes. If anyone wants to use the film clips rated in this study, one can request at rlzhou@nju.edu.cn for them. And you will be asked to confirm that it will be used solely for non-profit scientific research and that the database will not be reproduced or broadcasted in violation of international copyright laws.
